# Construction and Validation of a General Medical Image Dataset for Pretraining

**DOI:** 10.1007/s10278-024-01226-3

**Published:** 2024-08-15

**Authors:** Rongguo Zhang, Chenhao Pei, Ji Shi, Shaokang Wang

**Affiliations:** 1 Academy for Multidisciplinary Studies, Capital Normal University, 105 West Third Ring Road North, Haidian District, Beijing, China; 2 Institute of Advanced Research, Infervision, Beijing, China

**Keywords:** Deep learning, Transfer learning, Medical image classification and segmentation, Pretrained model

## Abstract

In the field of deep learning for medical image analysis, training models from scratch are often used and sometimes, transfer learning from pretrained parameters on ImageNet models is also adopted. However, there is no universally accepted medical image dataset specifically designed for pretraining models currently. The purpose of this study is to construct such a general dataset and validate its effectiveness on downstream medical imaging tasks, including classification and segmentation. In this work, we first build a medical image dataset by collecting several public medical image datasets (CPMID). And then, some pretrained models used for transfer learning are obtained based on CPMID. Various-complexity Resnet and the Vision Transformer network are used as the backbone architectures. In the tasks of classification and segmentation on three other datasets, we compared the experimental results of training from scratch, from the pretrained parameters on ImageNet, and from the pretrained parameters on CPMID. Accuracy, the area under the receiver operating characteristic curve, and class activation map are used as metrics for classification performance. Intersection over Union as the metric is for segmentation evaluation. Utilizing the pretrained parameters on the constructed dataset CPMID, we achieved the best classification accuracy, weighted accuracy, and ROC-AUC values on three validation datasets. Notably, the average classification accuracy outperformed ImageNet-based results by 4.30%, 8.86%, and 3.85% respectively. Furthermore, we achieved the optimal balanced outcome of performance and efficiency in both classification and segmentation tasks. The pretrained parameters on the proposed dataset CPMID are very effective for common tasks in medical image analysis such as classification and segmentation.

## Introduction

Transfer learning [1] is a deep learning method where a model trained for one task is used as the training start point for the model on another task. The pretrained model parameters from ImageNet [2] are usually used in various areas including medical images. The idea is that the pretrained models may have learned general features and representations from a large and diverse dataset, which can be beneficial for other related tasks. Although transfer learning based on ImageNet is widely applied [3–6], there is a domain gap between ImageNet dataset and the target dataset. The evaluation on two large medical image datasets [7] showed that transfer learning offered little benefit to performance and simple models can perform comparably to standard ImageNet models. However, small-scale medical imaging datasets are more prevalent in both scientific research and general applications. These datasets often contain a limited number of annotated samples due to the inherent challenge in medical imaging of the specialized and costly annotation. In such cases, transfer learning from pretrained models on large-scale datasets like ImageNet becomes a valuable approach [8], which helps to overcome the limitations of small-scale medical images and improves the performance of efficient analysis and diagnosis.

The work [9, 10] showed that models pretrained on grayscale ImageNet performed better in both speed and accuracy on X-ray image classification. An intuitive explanation could be that gray images have more similar features with medical images. The study [11] provided an effective pretraining method by using 2D radiographs which can outperform ImageNet pretraining. The work [12] proposed RadImageNet pretrained models to demonstrate better interpretability compared with ImageNet models especially for smaller radiologic datasets. These works all realized the difference between the pretraining domain and the target task domain and the importance of transfer learning based on pretrained models from medical images. However, there is still a lack of a universal medical imaging dataset for obtaining pretrained models that can be applied to small-scale medical imaging analysis tasks downstream. The research [13] presented that a large dataset is crucial for CNNs. By creating balanced sample spaces and using transfer learning, CNNs are better trained.

From the aspect of neural network structure, convolution neural networks (CNNs) have reigned for many years as the approach of medical image analysis. In recent years, Vision Transformers (ViTs) have become increasingly popular for image intelligent recognition [14, 15]. Transformers have outperformed CNNs on many vision tasks such as classification [16] and semantic segmentation [17] due to the attention mechanism. CNNs or ViTs usually perform worse when the data is scarce, so employing transfer learning is the typical solution.

In this study, we first build a medical image dataset by collection of several public medical image datasets, called CPMID for short, covering X-ray, CT, and MRI modalities. And then, we train the pretrained models on CPMID using Resnet [18] with different complexities and Vision Transformer. At last, we compared the experimental results on the other three publicly accessible small-scale medical image datasets by training from scratch, from the pretrained parameters on ImageNet, and from the pretrained parameters on CPMID. We also study the effects of different complexity neural network structures on transfer learning. Classification performance is measured on metrics such as model complexity, classification accuracy, and class activation heatmaps [19], and segmentation performance is measured in terms of pixel intersection-over-union (IOU) [20] averaged across the four classes.

The main contributions are as follows:

1. Based on several publicly medical image datasets, a medical domain dataset for pretraining and five pretrained models for transfer learning were constructed.
2. The effectiveness of these pretrained models from the proposed dataset was validated on classification and segmentation tasks.
3. On downstream medical image analysis, the results of training initialization with from scratch, with pretrained parameters from ImageNet, and with pretrained parameters from CPMID were compared, demonstrating that transfer learning based on the proposed dataset is the most effective.
4. The effectiveness of transfer learning was compared across network structures with different complexities, confirming that transfer learning based on pretrained models of the same domain with a simpler network structure yields the best results on smaller datasets.

These experiments and findings in this study provide some appropriate guidelines for using transfer learning on common medical image analysis. For better reproducibility of the experimental results, we will share the proposed dataset for pretraining, the pretrained models, and the training configuration files soon.

## Materials and Methods

**Fig. 1:**
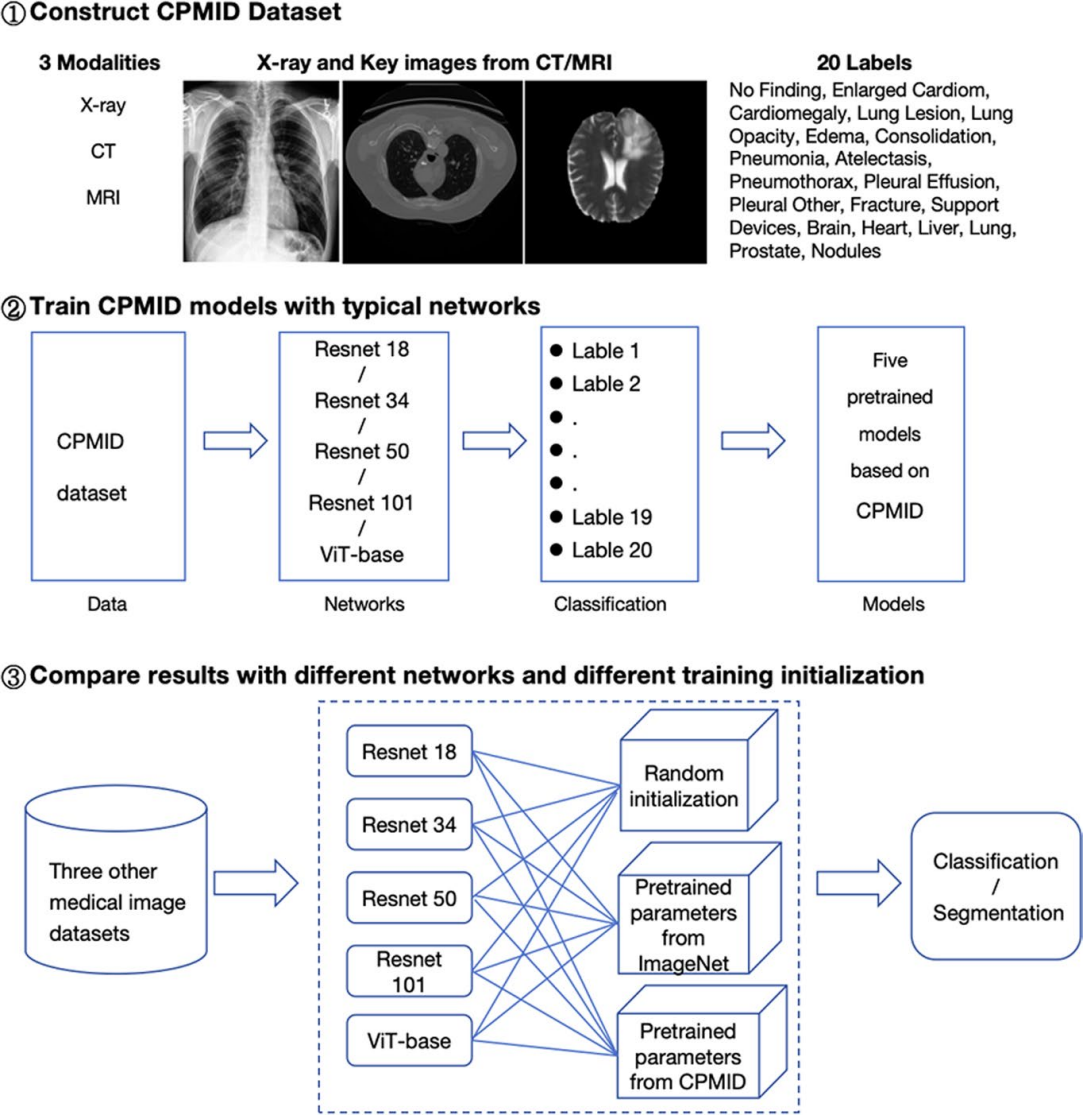
The overall framework of this study. CPMID = collection of several public medical image datasets, CT = computed tomography, MRI = magnetic resonance imaging, ViT = Vision Transformer

The entire process of this study primarily consists of three main components: construction of the pretraining dataset CPMID, training the pretrained models, and the results comparison of with different training initialization on three other medical image datasets. The overall framework is illustrated in Fig. 1.

### Construction of the Pretraining Dataset

We collected several larger public medical image datasets containing CheXpert dataset [21], Medical Segmentation Decathlon (MSD) dataset [22], and LIDC-IDRI dataset [23] to build the pretraining medical database CPMID. The following are how we get CPMID from these three open datasets. The CheXpert dataset contains 224,316 chest radiographs of 65,240 patients. The X-ray images are provided with 14 labels (no finding, enlarged Cardiom, cardiomegaly, lung lesion, lung opacity, edema, consolidation, pneumonia, atelectasis, pneumothorax, pleural effusion, pleural other, fracture, support devices) derived the corresponding radiology reports. These 14 categories of X-ray images serve as the primary components of CPMID.

This MSD dataset contains a total of 2633 three-dimensional images collected across multiple anatomies of interest, multiple modalities, and multiple sources. To facilitate the subsequent training task of classification, five organ categories (brain, heart, liver, lung, prostate) with a larger proportion in the images were selected. We chose the key image frames of transverse section from 3d volume images according to the segmentation mask. The selected five categories of CT/MR key images serve as an additional component of the CPMID database.

The dataset LIDC-IDRI contains lesion annotations from four experienced thoracic radiologists, which contains 1018 low-dose lung CTs from 1010 lung patients. Due to the relatively small proportion of the lung nodule lesions in the image, we cropped a region of 224*224 around the nodule approximately as the saved image. The cropped nodule images are the last part of the CPMID database.

Finally, the built medical database CPMID consists of 530,380 medical images from X-ray, CT, and MRI modalities. The summary table describing for pretraining dataset is listed in Table 1, containing the label name, the number of images per class, the type of imaging, and the respective spatial resolution. Although smaller in size compared to ImageNet, it basically covers common medical imaging modalities, major organs, and lesions.

**Table 1:** The summary table for describing the training dataset in CPMID

Class labels	Image number	Image type	Resolution
No finding	22,381	X-ray	390*320
Enlarged Cardiom	10,798	X-ray	390*320
Cardiomegaly	27,000	X-ray	390*320
Lung lesion	9186	X-ray	[320,390]*[320,390]
Lung opacity	105,581	X-ray	390*320
Edema	52,246	X-ray	390*320
Consolidation	14,783	X-ray	390*320
Pneumonia	6039	X-ray	[320,390]*[320,390]
Atelectasis	33,376	X-ray	[320,390]*[320,390]
Pneumothorax	19,448	X-ray	[320,390]*[320,390]
Pleural effusion	86,187	X-ray	[320,390]*[320,390]
Pleural other	3523	X-ray	[320,390]*[320,390]
Fracture	9040	X-ray	[320,390]*[320,390]
Support devices	116,001	X-ray	390*320
Brain	4788	MRI	240*240
Heart	372	MRI	320*320
Liver	2143	CT	512*512
Lung	465	CT	512*512
Prostate	216	MRI	256*256
Nodules	4708	CT	224*224

### Pretrained Models on CPMID

As shown in Fig. 1, following the construction of the CPMID database, we trained the 20-category classification model using several typical networks, respectively. For comparing the impact of network structures with different complexities on transfer learning, Resnet18, Resnet34, Resnet50, Resnet101, and Vision Transformer networks are chosen. The classic vision network architectures ranging from simple to complex are covered. The training procedure is implemented using MMClassification toolbox from the OpenMMLab [24] project based on PyTorch with two NVIDIA GeForce RTX 3090 GPU. For fair comparisons, we trained all models for 100 epochs with the consistent hyperparameters and model settings except the network structure itself. The batch size is 256, so there are 2000 iterations in one epoch. SGD optimizer is used with initial learning rate of 0.1, a weight decay of 0.0001, and momentum of 0.9.

The FLOPs and the size of params are usually used to demonstrate the complexity of a model. The two indicators of our used models for the input shape (3, 224, 224) are listed in Table 2. The numerical value in the fourth column represents the training time on CPMID for different network structures.

**Table 2:** The FLOPs and parameter sizes of different networks

Networks	FLOPs	Params	Training time on CPMID
Resnet18	1.82G	11.18 M	6 h 25 m 34 s
Resnet34	3.68G	21.29 M	9 h 54 m 32 s
Resnet50	4.12G	23.51 M	17 h 1 m 40 s
Resnet101	7.85G	42.50 M	27 h 46 m 27 s
ViT-base	16.86G	85.80 M	41 h 5 m 48 s

*FLOPs *floating point operations, *CPMID *collection of several public medical image datasets

**Fig. 2:**
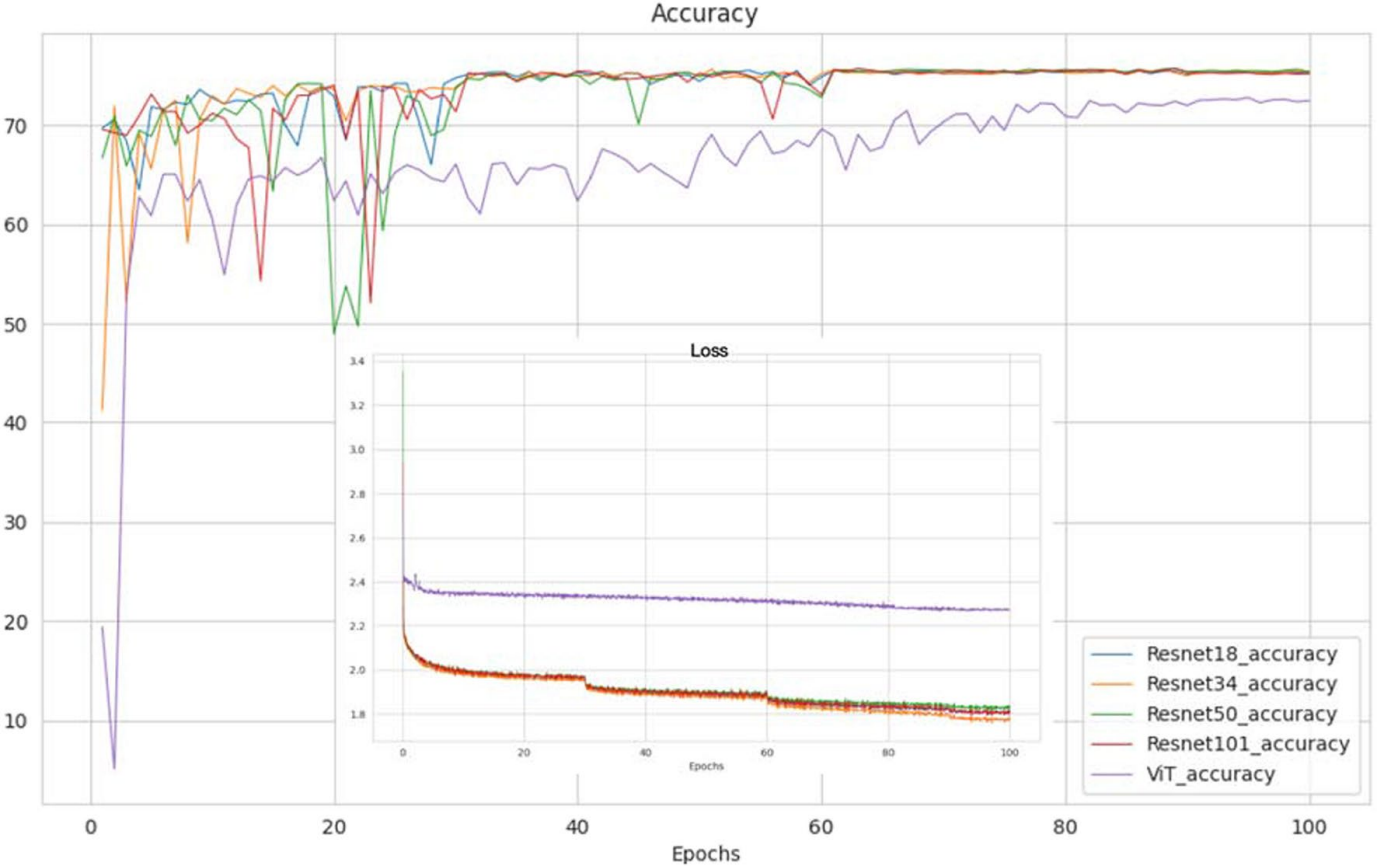
The validation accuracy curves during the training process by using different networks. ViT Vision Transformer

During the whole training process, we obtain a trained model at every epoch, along with the accuracy on the validation set. The curves in Fig. 2 show that the CNN series (Resnet18, Resnet34, Resnet50, Resnet101) consistently achieve high levels of accuracy, outperforming the used ViT model. We believe that it is ViT’s reliance on large size of data that has caused this result. We can see that within 100 epochs, the training models have all reached convergence. The models with the best performance on the validation set will be used as the pretrained models respectively for the five different network structures in subsequent comparisons.

### Validation Datasets

The public datasets ChestXRay2017 [25] and the tuberculosis (TB) dataset [26] are used in the classification validation task. ChestXRay2017 consists of 5856 X-ray images with 400–2000-pixel resolution. The images are divided into three categories: normal, bacterial pneumonia, and viral pneumonia. The number of images in each category is 1583, 2780, and 1493, respectively. TB dataset consists of two publicly available smaller datasets, MontgomeryCXRSet and ChinaCXRSet, released by the National Library of Medicine [27]. In the TB dataset, the number of images in the normal and tuberculosis categories is respectively 406 and 394. As listed in Table 3, the training and validation sets are divided according to the partitioning of the ChestXRay2017 datasets itself in the multiclass classification. And the TB dataset is divided into a 9:1 ratio for training and validation in binary classification experiment.

**Table 3:** The training set and validation set of two datasets for multiclass and binary classification

Classification	Types	Training set	Validation set
ChestXRay2017-multiclass	Normal	1349	234
	Bacterial pneumonia	2538	242
	Viral pneumonia	1345	148
TB-binary	Normal	366	40
	Tuberculosis	355	39

*TB* tuberculosis

To demonstrate the general applicability of our proposed method within the medical imaging domain, we introduced an additional validation dataset that is completely distinct from the images used for pretraining in the construction dataset. BreastMNIST [28] contains 780 breast ultrasound images, which simplify the task into binary classification by combining normal and benign as positive and classifying them against malignant as negative. The source images are resized into 224 × 224.

In the segmentation task, we used the dataset from CHAOS challenge [29], which aims to segment four abdominal organs (liver, right kidney, left kidney, and spleen) from MRI data. Since the test set is not publicly available, we can only use the available training data for experimentation. The datasets are acquired by a 1.5 T Philips MRI, which produces 12-bit DICOM images having a resolution of 256 × 256. The ISDs vary between 5.5 and 9 mm (average 7.84 mm), *x*–*y* spacing is between 1.36 and 1.89 mm (average 1.61 mm), and the number of slices is between 26 and 50 (average 36).

### Comparison Module

In the comparison phase shown, we first train classification and segmentation models for comparison, with each model being trained on five different network structures under three different initialization conditions, namely, training from scratch (random initialization), the pretrained parameters from ImageNet, and the pretrained parameters from CPMID. Then, the trained models are individually applied to the validation data to obtain their respective results.

To compare the performance impact of transfer learning on different models, the training and validation conditions for the three methods are kept consistent, except for the difference in training starting points. For each network associated with an initialization method, the trained model that performs best on the test set will be chosen for comparison experiments.

## Results

We carried out a comprehensive set of experiments on external downstream tasks to evaluate the performance of models that were trained from scratch, from pretrained parameters on ImageNet, or from pretrained parameters on CPMID. The comparison experiments contain three classification tasks and one segmentation task. As described in the method section, we have trained 39 classification models and six segmentation models totally for the external validation tasks.

### Classification Results

**Fig. 3:**
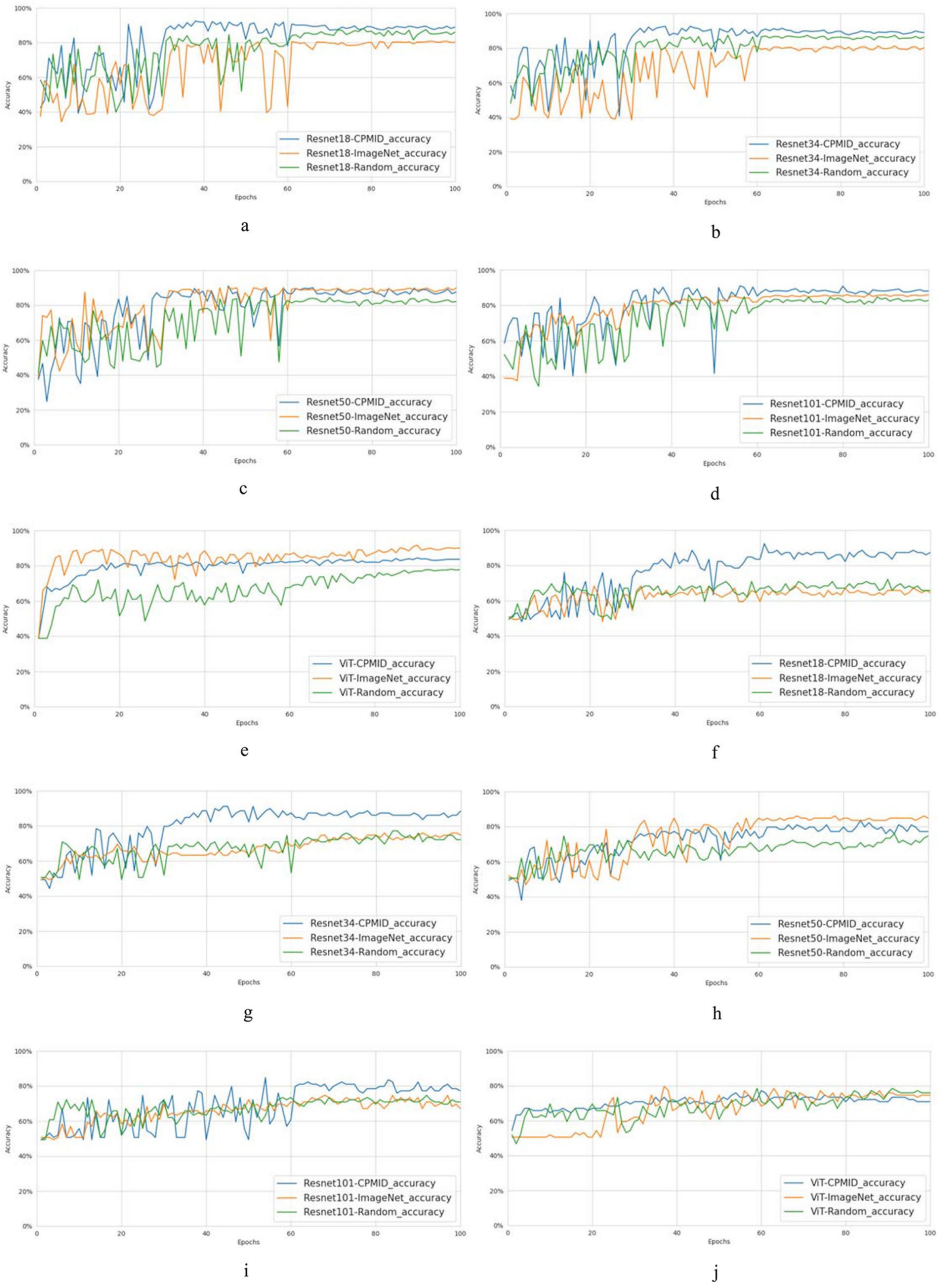
The classification performance comparison of three different training initializations with the five different networks, Resnet18, Resnet34, Resnet50, Resnet101, and ViT, respectively. The curves **a**–**e** show the validation results of the models at different training epochs on the ChestXRay2017 dataset. The curves **f**–**j** show the validation results of the models at different training epochs on the TB dataset. ViT Vision Transformer, CPMID collection of several public medical image datasets, TB tuberculosis

In the three classification experiments, we applied various networks and different initialization starting points to train the models separately. There are five different networks: Resnet18, Resnet34, Resnet50, Resnet101, and ViT. The three different training initialization methods include Random, pretrained parameters from ImageNet, and pretrained parameters from CPMID. There are a total of 39 classification models and 39 corresponding test results for the three experiments totally. To facilitate comparison, each model was trained for 100 epochs without any special optimization. The first two classification experimental results are illustrated in Fig. 3. The horizontal axis in the figures represents the trained model associated with different epochs, and the vertical axis denotes the corresponding test accuracy. From these figures, it can be observed that neural networks with simpler structures, such as Resnet18 and Resnet34, demonstrate the optimal performance in the two classification experiments when the CPMID pretrained parameters are used. Furthermore, as the network architecture grows in complexity, the efficacy of the method utilizing ImageNet pretrained parameters progressively enhances. Nevertheless, it remains inferior to the optimal outcome achieved with CPMID, especially in balancing efficiency and accuracy.

The more detailed comparison of results can be found in Tables 4 and 5. We use the accuracy and class-weighted accuracy metrics to measure different methods. The best validation accuracy values during the 100 epochs with different networks and initialization strategies are listed. We can observe that the method employing CPMID pretrained parameters with Resnet34 yielded the best result of 0.9263 and 0.9176 in the multiclassification task on ChestXRay2017 and achieved the highest values of 0.9241 and 0.9234 in the binary classification task on the TB dataset using Resnet18. As listed in Table 6, the classification metrics of sensitivity, specificity, accuracy, and class-weighted accuracy are compared for the external ultrasound dataset. It can be seen that the validation results based on the pretraining dataset constructed in this study are the best, and there is a better balance between sensitivity and specificity. Generally, using a simpler network structure for initial training with CPMID pretrained parameters yields the best results, overall surpassing the initial training based on ImageNet pretrained parameters. Notably, the average accuracy based on CPMID outperformed ImageNet-based results by 4.30%, 8.86%, and 3.85% on the three validation datasets respectively. Therefore, for some medical image classification tasks of a similar scale to these three datasets in this study, it is recommended to use transfer learning from the proposed CPMID pretrained parameters with a relatively simple network. Through this method, the model can be trained efficiently and achieve very good results. The training and validation are also implemented based on PyTorch with two NVIDIA GeForce RTX 3090 GPU.

**Table 4:** For ChestXRay2017, the comparison of classification accuracy, class-weighted accuracy, and F1 score with different networks and initialization strategies

Networks	Random initialization	Pretrained parameters from ImageNet	Pretrained parameters from CPMID
Resnet18	0.8814, 0.8700, 0.8817	0.8077, 0.7982, 0.8069	0.9247, 0.9160, 0.9246
Resnet34	0.8750, 0.8621, 0.8755	0.8125, 0.8058, 0.8123	**0.9263, 0.9176, 0.9263**
Resnet50	0.8590, 0.8383, 0.8568	0.9022, 0.8794, 0.8999	0.9103, 0.8924, 0.9087
Resnet101	0.8590, 0.8364, 0.8566	0.8622, 0.8423, 0.8610	0.9103, 0.8949, 0.9097
ViT-base	0.7804, 0.7594, 0.7779	0.9167, 0.9110, 0.9172	0.8445, 0.8365, 0.8388

*ViT *Vision Transformer

Values in bold indicate the highest values of the corresponding performance metrics

**Table 5:** For TB dataset, the comparison of classification accuracy, class-weighted accuracy, and F1 score with different networks and initialization strategies

Networks	Random initialization	Pretrained parameters from ImageNet	Pretrained parameters from CPMID
Resnet18	0.7215, 0.7205, 0.6944	0.6835, 0.6821, 0.6377	**0.9241, 0.9234, 0.9189**
Resnet34	0.7722, 0.7712, 0.7500	0.7595, 0.7577, 0.7164	0.9114, 0.9106, 0.9041
Resnet50	0.7595, 0.7580, 0.7246	0.8608, 0.8596, 0.8451	0.8354, 0.8353, 0.8312
Resnet101	0.7468, 0.7446, 0.6875	0.7468, 0.7455, 0.7143	0.8481, 0.8481, 0.8462
ViT-base	0.7848, 0.7824, 0.7302	0.7975, 0.7958, 0.7647	0.7722, 0.7702, 0.7273

*TB* tuberculosis, *CPMID* collection of several public medical image datasets

Values in bold indicate the highest values of the corresponding performance metrics

**Table 6:** For the BreastMNIST ultrasound dataset, the comparison of sensitivity (Se), specificity (Sp), accuracy (Acc), class-weighted accuracy (WAcc) and F1 score with different networks and initialization strategies

Network metrics	Random initialization					Pretrained parameters from ImageNet					Pretrained parameters from CPMID				
	Se	Sp	Acc	WAcc	F1	Se	Sp	Acc	WAcc	F1	Se	Sp	Acc	WAcc	F1
Resnet18	0.965	0.476	0.833	0.721	0.894	0.983	0.143	0.756	0.563	0.855	0.842	0.714	0.808	0.778	0.865
Resnet34	0.965	0.500	0.840	0.733	0.898	0.956	0.333	0.789	0.645	0.869	0.921	0.738	**0.872**	**0.830**	0.913
Resnet50	0.974	0.262	0.782	0.618	0.867	0.947	0.667	0.872	0.807	**0.915**	0.886	0.762	0.853	0.824	0.898

Values in bold indicate the highest values of the corresponding performance metrics

**Fig. 4:**
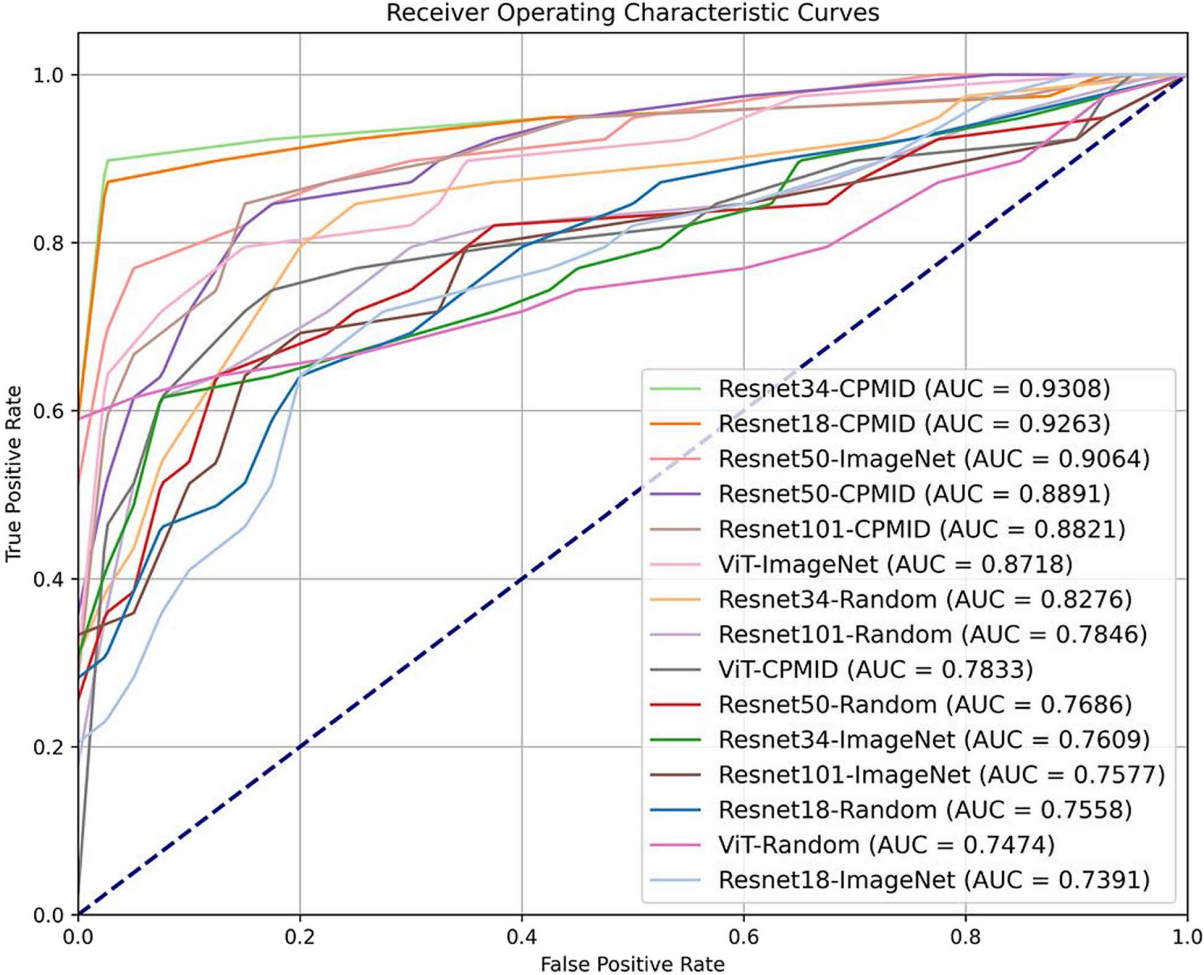
The ROC curves of all the binary classifiers on the TB dataset. ViT Vision Transformer, CPMID collection of several public medical image datasets

To more intuitively compare the performance of various binary classifiers on the TB dataset, we have created the corresponding receiver operating characteristic (ROC) curves as shown in Fig. 4. The names in the legend are composed of the network architecture and the initialization conditions. We can see that the simpler network architectures such as Resnet18 and Resnet34, which underwent transfer learning based on CPMID constructed in this paper, achieved the best results. The top six methods in terms of performance are all based on transfer learning, and their results overall outperform those of other methods significantly.

**Fig. 5:**
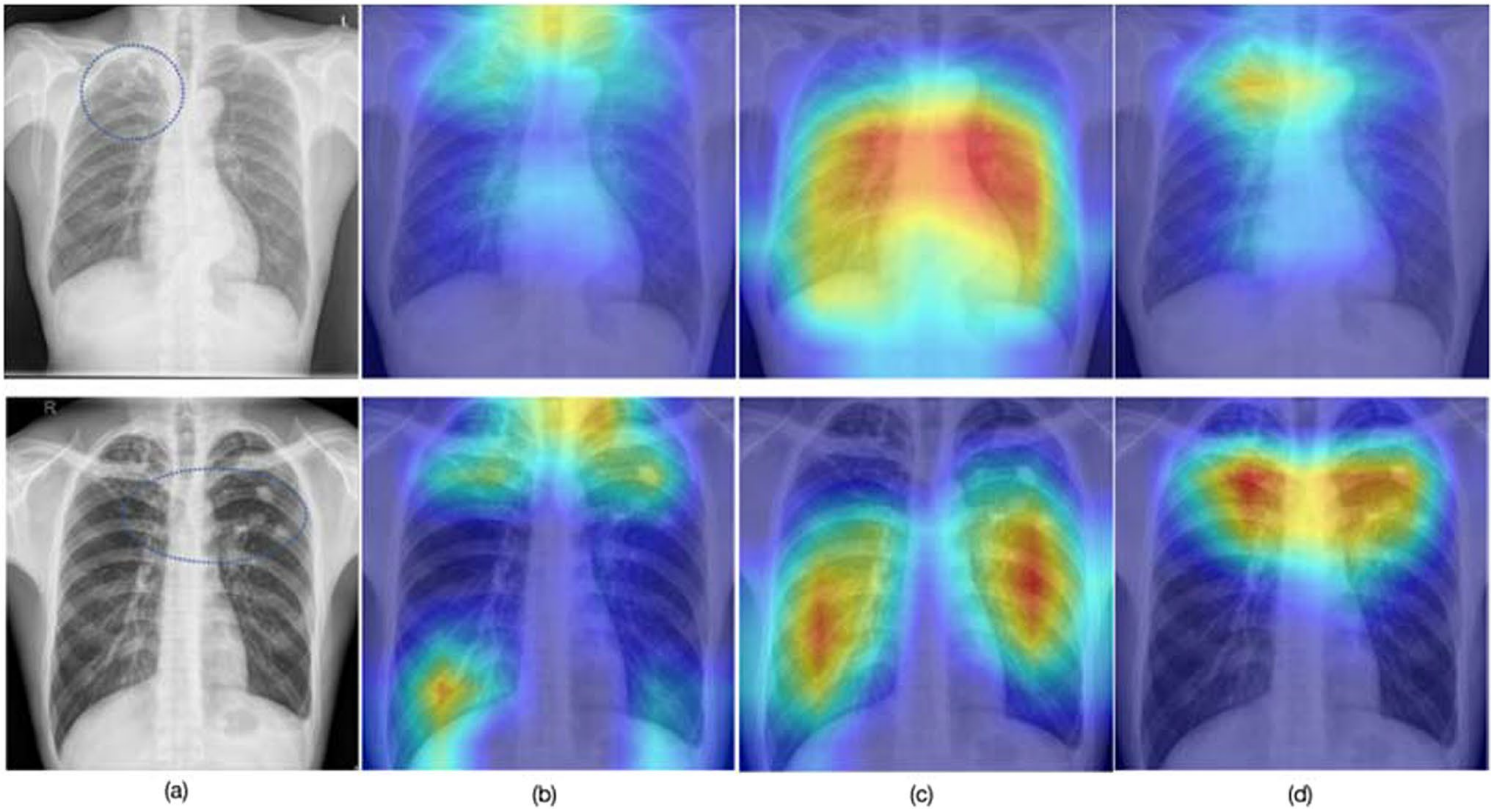
Visualizations of the gradient-weighted class activation maps. **a** The two original X-ray images with dashed circles marking the approximate location of the TB lesion. **b** The GradCAMs based on random initialization method. **c** The GradCAMs based on ImageNet pretrained method. **d** The GradCAMs based on the proposed CPMID pretrained method. GradCAMs gradient-weighted class activation maps, CPMID collection of several public medical image datasets

Furthermore, we compared the effectiveness of transfer learning from the perspective of heatmap activations. The ground-truth positions of the TB lesions in the validation dataset were indicated by a radiology doctor. Figure 5 shows the visualizations of the gradient-weighted class activation maps (GradCAMs) [30] on two representative examples of tuberculosis images. The norm layer of the Resnet18-based model’s last block is used to get GradCAM. By aligning the location of the lesion with that of the original image, the heatmap generated by the proposed CPMID pretrained method exhibits higher accuracy and better focus compared to the other two methods.

### Segmentation Results

For the segmentation experiments, the 20 cases of T1-weighted sequences were randomly divided into three parts for conducting threefold cross-validation in this experiment. Each case corresponds to a series of DICOM images belonging to a single patient. The experiments were based on the DeeplabV3 [31] network which uses Resnet as its backbone network and cross-entropy as the loss function to realize image semantic segmentation. In our research, ResNet-50 and ResNet-101 were used as backbone networks, and model parameter initialization was performed from scratch, ImageNet pretrained weights, and CPMID pretrained parameters respectively. The metrics for evaluations are Intersection over Union (IoU) for the four organs, including the liver, right kidney, left kidney, and spleen, and mean IoU (mIoU) which can comprehensively reflect the segmentation performance of the model on the four categories. All the results are listed in Table 7.

**Table 7:** The segmentation IoU results of four abdominal organs and mIoU with different networks and initialization strategies

Initialization	Network	Liver	Right kidney	Left kidney	Spleen	mIoU
Random initialization	Resnet50	0.8902	0.7834	0.7518	0.7352	0.7902
	Resnet101	0.8628	0.5754	0.4499	0.6438	0.6330
Pretrained parameters from ImageNet	Resnet50	0.8969	0.8008	0.7589	0.7596	0.8041
	Resnet101	0.9056	0.8104	0.7629	0.7909	**0.8167**
Pretrained parameters from CPMID	Resnet50	0.8998	0.8078	0.7594	0.7973	**0.8161**
	Resnet101	0.8977	0.7872	0.7528	0.7909	0.8072

*IoU* Intersection over Union, *mIoU* mean Intersection over Union

Firstly, the segmentation results based on pretrained parameters are all better than those with random initialization; secondly, the average result of two networks based on CPMID 0.8117 is slightly better than that based on ImageNet 0.8104, but the difference is not significant. Segmentation is a more complex task than classification. Although CPMID has the advantage of a similar domain, ImageNet has the advantage in terms of quantity. Therefore, the results of pretrained parameters based on the two datasets are not significantly different. Nevertheless, we can still observe that the mIoU values achieved by CPMID pretrained method with Resnet50 are highly comparable with ImageNet pretrained method with Resnet101, despite the fact that the Resnet50 model has a much lower complexity than the Resnet101 model. Therefore, compared to pretrained models based on ImageNet, using a pretrained model based on CPMID can significantly reduce the time for training and inference while ensuring accuracy.

## Discussion

ImageNet-pretrained models have been widely used in medical image analysis though there is an obvious domain gap between natural images and medical images. To the best of our knowledge, this is the first work to use multiple networks and different pretrained parameters to assess effective of transfer learning on small medical datasets comprehensively. In this study, the proposed dataset CPMID was first built by collection of several common open medical imaging datasets, which include various common radiological medical imaging modalities. We utilize the lesions and tissue organs contained within the images as category labels. The built progress of CPMID provides insights for the construction of medical imaging database with a large amount of dataset like ImageNet. This study conducted extensive experiments to evaluate the applicability and value of pretrained models on small-scale medical imaging datasets. In the assessment stage, a series of extensive classification and segmentation experiments were conducted on three other small medical image datasets, using three different training initialization methods and five different networks.

By comparing the three training initialization methods in downstream tasks, we found that training from pretrained parameters is much better than from scratch on small medical datasets. Especially, utilizing pretrained parameters from CPMID achieved the best balance result in terms of performance and efficiency. Additionally, transfer learning based on CPMID pretrained parameters also offers better interpretability by heatmaps in the classification task. Hence, for small-scale medical image dataset, transfer learning should be utilized regardless of a classification or segmentation task, which is significantly better than starting training from scratch. For all tasks, the model training from CPMID pretrained parameters can yield highly satisfactory results when using simpler network architectures. For complex networks such as ViT and complex tasks such as segmentation, the use of ImageNet-based pretrained parameters is also beneficial.

At the same time, when considering transfer learning methods solely based on ImageNet pretrained weights, our research also found that as the network structure grows more intricate, the performance enhances incrementally, particularly when using the ViT architecture for multiclass classification tasks on the ChestXRay2017 dataset. A similar observation was noted in the segmentation experimental tasks. These above observations are explainable. For deep learning applications, both complex models and tasks require a substantial amount of data for fitting. The visualizations of the gradient-weighted class activation maps demonstrated the utility of the proposed dataset in the realm of explainable artificial intelligence (XAI). Establishing trustworthiness in XAI is a critical research endeavor, particularly within the medical domain [32–34]. Furthermore, we contend that large-scale pretraining may offer a potential pathway to enhancing the interpretability of models. However, it is worth noting that the effectiveness of transfer learning is not solely dependent on the complexity of the network architecture and the similarity between the pretraining dataset and the target task dataset. Other factors, such as the size of the target dataset and the availability of labeled data, also play crucial roles. Gathering a medical imaging dataset larger than CPMID for pretraining models is an important extension of the research. What’s more, fine-tuning large visual models (LVM) [35] on downstream small-scale datasets may be also a potential avenue for future research.

## Data Availability

The datasets that support the findings of this study are available from the corresponding author upon request.
